# Vitamin D, Hypercalciuria and Kidney Stones

**DOI:** 10.3390/nu10030366

**Published:** 2018-03-17

**Authors:** Emmanuel Letavernier, Michel Daudon

**Affiliations:** 1 Department: UMR S 1155, Sorbonne Université-UPMC Paris 06, F-75020 Paris, France; michel.daudon@aphp.fr; 2 Institut National de la Santé et de la Recherche Médicale (INSERM), UMR S 1155, F-75020 Paris, France; 3 Explorations Fonctionnelles Multidisciplinaires, AP-HP, Hôpital Tenon, F-75020 Paris, France

**Keywords:** vitamin D, kidney stones, calcium, phosphate

## Abstract

The estimated lifetime risk of nephrolithiasis is growing nowadays, and the formation of kidney stones is frequently promoted by hypercalciuria. Vitamin D, and especially its active metabolite calcitriol, increase digestive calcium absorption—as urinary calcium excretion is directly correlated with digestive calcium absorption, vitamin D metabolites could theoretically increase calciuria and promote urinary stone formation. Nevertheless, there was, until recently, low evidence that 25-hydroxyvitamin D serum levels would be correlated with kidney stone formation, even if high calcitriol concentrations are frequently observed in hypercalciuric stone formers. Low 25-hydroxyvitamin D serum levels have been associated with a broad spectrum of diseases, leading to a huge increase in vitamin D prescription in the general population. In parallel, an increased frequency of kidney stone episodes has been observed in prospective studies evaluating vitamin D alone or in association with calcium supplements, and epidemiological studies have identified an association between high 25-hydroxyvitamin D serum levels and kidney stone formation in some groups of patients. Moreover, urinary calcium excretion has been shown to increase in response to vitamin D supplements, at least in some groups of kidney stone formers. It seems likely that predisposed individuals may develop hypercalciuria and kidney stones in response to vitamin D supplements.

## 1. Introduction

Urolithiasis is a growing public health problem worldwide, with an estimated lifetime risk of around 10% of the population in some countries [1,2]. This increase has been attributed, at least in part, to dietary environmental factors, such as increased salt and protein intakes, which are responsible for hypercalciuria, one of the main determinants of calcium-dependent kidney stone formation [3]. Kidney stones made of calcium oxalate and, to a lesser extent, calcium phosphate represent more than 80% of total stones in Western countries [4,5]. Moreover, we have observed an increased proportion of stones developed on Randall’s plaques during the last decades [6]. These plaques are calcium phosphate deposits appearing in kidney interstitial tissue whose formation is associated with hypercalciuria, and frequently constitutes the first step in stone formation [7,8,9,10,11]. Whether vitamin D supplements or high 25-hydroxyvitamin D serum levels may increase urinary calcium excretion and promote kidney stone formation has been debated. Recent studies have highlighted that some subgroups of patients may be at risk of developing kidney stones when receiving vitamin D supplements, mainly cholecalciferol [12,13,14]. In addition, recent meta-analyses have highlighted that 25-hydroxyvitamin D serum levels are higher in kidney stone patients affected by hypercalciuria and kidney stones [15,16]. These observations should sound a warning, especially in clinical settings where a vitamin D supplement benefit has not been established. We review hereafter the seminal studies that established a link between vitamin D metabolites (especially calcitriol) and hypercalciuria or kidney stone formation in humans, the data resulting from animal experimental studies and the epidemiological and interventional studies dedicated to vitamin D and kidney stones, and discuss the potential link between vitamin D and Randall’s plaque formation.

## 2. Methods

Literature Search and Study Selection:

NIH-PubMed/Medline, ISI-Web of Science and Cochrane Library were searched to identify relevant studies reporting the relationship between vitamin D, hypercalciuria and kidney stones. The search was performed several times between September 2017 and November 2017. The initial search process was designed to find all relevant published original articles without limitation by year or language. Detailed search terms were (“stone” OR “kidney stone” OR “urolithiasis” OR “nephrolithiasis” OR “calculus” OR “Randall’s plaque” OR “hypercalciuria” or “urine calcium”) AND (“vitamin D” OR “calcitriol” OR “cholecalciferol” OR “25-hydroxyvitamin D” OR “25-hydroxycholecalciferol” OR “1,25-dihydroxyvitamin D” OR “Vitamin D receptor” OR “(Vitamin D receptor) VDR”). Two authors (E.L. and M.D.) screened citations returned from the search strategy to identify eligible studies.

Conference abstracts were not included, but all peer-reviewed studies were considered and discussed in the review. In addition, some significant studies dedicated to Randall’s plaque and to the biological role of vitamin D are also cited and discussed in this review.

## 3. Vitamin D and Kidney Stones: An Old Story

### 3.1. Calcitriol (1,25-Dihydroxyvitamin D) and Absorptive Hypercalciuria

The main component of kidney stones is calcium oxalate, and, to a lesser extent, calcium phosphate. Increased urinary calcium excretion or hypercalciuria is one of the main risk factors promoting calcium kidney stone formation. Early studies have demonstrated increased intestinal absorption of calcium in most cases of idiopathic hypercalciuria, defining absorptive hypercalciuria [17,18]. Intestinal calcium absorption depends on the calcium intraluminal concentration (paracellular absorption), but the main factor responsible for transcellular calcium absorption is 1,25-dihydroxyvitamin D or calcitriol, the active form of vitamin D [19]. Vitamin D, whether produced in the skin from 7-dehydrocholesterol or absorbed from the diet or supplements must actually be activated as 25-hydroxyvitamin D in the liver and then as calcitriol in the kidneys to exert its biological effects.

Calcitriol binds the vitamin D receptor (VDR) in enterocytes and increases calcium transport across digestive epithelia through the gatekeeper transient receptor potential vanilloid 6 (TRPV6) transporter [20].

Calcitriol also binds VDR in parathyroid cells, decreasing parathyroid hormone (PTH) synthesis. PTH increases calcium influx through the gatekeeper TRPV5 in distal tubular kidney cells. Thus, PTH decrease by calcitriol may be associated with increased urine calcium excretion [21].

A correlation between calcitriol serum levels and net intestinal calcium absorption has been clearly demonstrated [22]. In pathological settings, a positive correlation between urine calcium excretion and calcitriol has been evidenced in both sarcoidosis and primary hyperparathyroidism, two diseases responsible for increased calcitriol production by granuloma cells and by the kidneys, respectively [23,24].

Several groups reported, at the same time, the presence of high calcitriol levels in idiopathic hypercalciuric stone formers, suggesting that absorptive hypercalciuria might result from high calcitriol levels [25,26,27]. There is a correlation between calcitriol serum levels and urine calcium excretion in stone formers but also in control patients [28,29,30]. The administration of ketoconazole (a drug decreasing calcitriol formation) to patients affected by absorptive hypercalciuria, reduced calciuria efficiently, evidencing the direct role of calcitriol on urinary calcium excretion [31]. Nevertheless, some patients did not lower calciuria in response to ketoconazole, suggesting that other mechanisms, independent of vitamin D signalling, may be involved in some groups of patients.

### 3.2. Why Do Calcium Stone Formers Have Higher Calcitriol Levels?

The mechanisms responsible for high calcitriol levels in kidney stone former populations are still under debate. It has been proposed that, at least in some patients, low serum phosphate levels could increase calcitriol production in the proximal tubules of the kidneys [25]. Recently, Schlingmann et al. provided evidence that young patients affected by *SLC34A1* mutations coding for the NPT2a phosphate transporter have increased calciuria, related to increased calcitriol serum levels, due to fibroblast growth factor-23 suppression [32]. Other groups identified that high protein intakes and glomerular hyperfiltration are associated with the origin of the high calcitriol levels observed in patients affected by urolithiasis [33]. More recently, it has been evidenced that some cases of idiopathic infantile hypercalcemia are due to mutations of *CYP24A*1, the enzyme encoding 25-hydroxyvitamin D 24-hydroxylase, the key enzyme of calcitriol degradation [34]. Despite suppressed PTH levels, these children have abnormally high calcitriol levels which have been linked to increased digestive calcium absorption, hypercalcemia and hypercalciuria. Interestingly, recent studies have confirmed that calcitriol serum levels were increased in calcium stone formers compared to controls and demonstrated that stone formers had a lower serum 24,25-hydroxyvitamin D/25-hydroxyvitamin D ratio compared to controls [35]. *CYP24A*1 polymorphisms have been described in kidney stone formers [36]. It may therefore be hypothesized that a relative 25-hydroxyvitamin D 24-hydroxylase deficiency would be at the origin of high calcitriol levels in some kidney stone formers, but further studies are needed to confirm this hypothesis.

### 3.3. Sensitivity to Vitamin D and Kidney Stones

Many patients affected by absorptive hypercalciuria and kidney stones have calcitriol serum levels within the normal range. It has been hypothesized that these patients are more “sensitive” to vitamin D, and genetic studies suggested have a linkage between the *VDR* gene locus coding for the vitamin D receptor and idiopathic calcium stone formation [37]. Some groups identified VDR polymorphisms associated with kidney stone formation, but these results have not been confirmed in large populations [38,39,40]. It seems unlikely that the most frequent VDR polymorphisms play an important role in kidney stone formation [41]. It has also been proposed that VDR could be more expressed in kidney stone formers’ tissues, and a high VDR expression has been shown in hypercalciuric patients’ leucocytes [42]. In contrast, another group did not provide evidence for VDR overexpression in patients affected by hypercalciuria and urolithiasis [43]. Since vitamin D signalling depends on VDR, but also other transcription factors such as retinoid X receptors, other pathways may explain a “higher” sensitivity to vitamin D, but the underlying mechanisms remain unknown. At last, vitamin D metabolites may also increase phosphate intestinal absorption [44,45]. The roles of cholecalciferol and calcitriol in calcium phosphate stone formation remain to be determined.

## 4. Vitamin D and Kidney Stones: Lessons from Animal Models

Among the few animal models of kidney stone formation, the most interesting is certainly the genetic hypercalciuric stone-forming rat (GHS). This model has been obtained by inbreeding the most hypercalciuric progeny of successive generations of Sprague–Dawley rats [46,47]. When fed on a standard diet, these rats have a dramatically higher urinary calcium excretion than controls and develop kidney stones made of calcium phosphate, or calcium oxalate with the addition of hydroxyproline to the diet [48]. As in humans, hypercalciuria is a polygenic trait [49]. This rat model is essential for addressing the pathophysiology of hypercalciuria. There is dramatically increased intestinal calcium absorption in GHS rats but also increased bone resorption and reduced renal tubular calcium reabsorption [50,51,52]. These rats have increased biological activity of VDR in the bones and intestines and an increased VDR expression in the intestines, bones and kidneys. Calcitriol administration to GHS rats exacerbates calciuria by increasing intestinal calcium absorption but also bone resorption [49,50,51]. These observations support the role of VDR in human hypercalciuria, but also the potential roles of calcitriol and VDR in bone demineralization which frequently affects kidney stone formers [52,53].

When wild-type Sprague–Dawley rats were exposed to injections of cholecalciferol every 3 weeks, they developed hypercalciuria and tiny calcium phosphate kidney stones [54]. The administration of calcium at high concentration in drinking water did not promote the formation of significant stones. In contrast, the synergistic administration of calcium and cholecalciferol promoted the development of large stones, addressing the risk of calcium and cholecalciferol co-administration, at least in this model. Calcium oral intake, at least in normal ranges, is not a risk factor for kidney stones and may even be protective through the limitation of digestive oxalate absorption [55]. Whether the combined administration of calcium and cholecalciferol to humans may promote kidney stone formation is supported by interventional studies described hereafter.

## 5. Vitamin D Serum Levels and Vitamin D Prescription: A Link with Kidney Stones?

Since calcitriol increases digestive calcium absorption and, at least temporarily, serum calcium levels, it should necessarily increase urine calcium excretion to maintain calcium homeostasis (by increasing the calcium filtration load and stimulating the renal calcium sensing receptor). The prescription of cholecalciferol or analogous treatments increases circulating levels of 25-hydroxyvitamin D, which may act with low affinity on VDR or be transformed into calcitriol, with a higher affinity to VDR [19]. The production of calcitriol is fortunately limited by parathyroid hormone synthesis suppression, through calcium sensing receptors and calcitriol signalling in parathyroid cells. Since parathyroid hormone promotes renal calcium handling in the distal tubules, its suppression may also increase urinary calcium excretion.

Although there is a large consensus that high calcitriol levels increase urine calcium and kidney stone formation, whether serum 25-hydroxyvitamin D circulating levels or widespread vitamin D prescription could influence kidney stone formation is still debated.

## 6. Observational Studies

Some studies have shown a positive association between urinary calcium excretion and 25-hydroxyvitamin D serum levels in adult stone formers [56,57]. Other authors did not find a relationship between 25-hydroxyvitamin D and urine calcium excretion or prevalent kidney stone disease. In the National Health Nutrition Examination Survey (NHANES) III cross sectional study, high serum 25-hydroxyvitamin D concentrations were not associated with prevalent kidney stones (reported history or nephrolithiasis) [58]. A retrospective study performed in 169 patients with nephrolithiasis did not show a relationship between serum 25-hydroxyvitamin D level and 24-h urine calcium excretion [59].

In a prospective analysis of 193,551 participants in the Health Professionals Follow-up Study (HPFS) and Nurses’ Health Studies (NHS) I and II, performed by Ferraro et al. there was no statistically significant association between vitamin D intake and risk of stones in the HPFS and the NHS I groups but potentially a higher risk in the NHS II group (Hazard Ratio 1.18, 95% Confidence Interval 0.94, 1.48, *p* for trend = 0.02) [60]. Of note, the NHS II study has been performed more recently and women included in the NHS II study had a daily intake of vitamin D (mainly due to supplementation) that was much more significant than in the previous studies. It may be hypothesized that this increase in vitamin D intake may have enhanced stone risk in this specific cohort.

Although the role of 25-hydroxyvitamin D serum levels in kidney stone formation has been discussed, the role of calcitriol is not a matter of debate. For instance, Taylor et al. compared calcium and phosphorus regulatory hormones and the risk of incident symptomatic kidney stones in a case-control study including 356 incident stone formers and 712 controls [61]. Baseline plasma levels of 25-hydroxyvitamin D were similar in both groups but higher plasma calcitriol levels were independently associated with a higher risk of symptomatic stones. Interestingly, several studies did not find an association between urinary calcium excretion and 25-hydroxyvitamin D serum levels when taking into consideration all stone formers, but identified a strong correlation when considering hypercalciuric stone formers only [29,62]. This point is critical and highlights that patient phenotype and kidney stone analysis should be assessed cautiously. Actually, vitamin D metabolism influences urinary calcium excretion, but all kidney stones are not calcium-dependent. Although calcium oxalate is the main component of 60 to 80% of all urinary calculi, there is strong evidence that calcium oxalate stones may result from hypercalciuria but also from hyperoxaluria and/or low diuresis, sometimes in the absence of metabolic disorder [4,63,64,65].

A recent meta-analysis based upon six case-control studies and one randomized controlled trial reported in the literature investigated the relationship between circulating 25-hydroxyvitamin D and the risk of stone formation [15]. Data were available for 451 kidney stone formers and 482 controls. The results provided evidence that kidney stone formers had significantly higher levels of 25-hydroxyvitamin D than controls, both in European and Asian populations. Finally, in another meta-analysis, Hu et al. investigated the association between circulating vitamin D levels and urolithiasis; twenty-two observational studies involving 23,228 participants were included [16]. Among them, 19,718 were controls and 3510 were stone formers. Within the latter group, more precise distinction was made regarding calcium excretion. The main conclusion of the meta-analysis was that calcitriol was significantly increased in stone formers compared to controls while 25-hydroxyvitamin D was similar in both groups. However, hypercalciuric stone formers had significantly higher calcitriol levels but also higher 25-hydroxyvitamin D serum levels than normocalciuric patients and controls.

## 7. Interventional Studies

Although 25-hydroxyvitamin D serum levels may be associated with kidney stones, at least in hypercalciuric patients, whether vitamin D supplementation may increase the risk of stone formation is an open question.

A large placebo controlled study provided evidence that hypercalciuria and hypercalcemia frequently occur in patients receiving both vitamin D and calcium supplementation [66].

Some randomized controlled studies have reported an increased risk of kidney stones as an adverse event resulting from the administration of vitamin D associated with calcium supplementation [12,13]. Actually, most interventional studies on vitamin D2 and vitamin D3 (cholecalciferol) supplementation (and thus meta-analyses on this topic) have participants randomized to both calcium and vitamin D supplementation, making it difficult to separate the individual impact of each component. Of note, calcium intake is, by itself, not a risk factor for kidney stone formation, and an inverse relationship between calcium intakes and stone risk has been described [55]. It seems, therefore, likely that both calcium and cholecalciferol or other vitamin D metabolite administration, as are usually prescribed to many patients, exert a synergistic risk of kidney stone formation. It should be emphasized that calcium from dietary sources may have a different impact on urine calcium excretion than calcium from supplemental sources (pills) [67].

Cochrane meta-analyses reported a significant increased risk of kidney stones (+17%) from vitamin D and vitamin D analogues [68,69]. The largest meta-analysis by Malihi et al. concluded that vitamin D2 and D3 supplementation resulted in changes in calcium metabolism, with increased risks of hypercalcemia and hypercalciuria, but no increase in the risk of reported kidney stones [14]. This analysis demonstrated that natural vitamin D by itself, even when calcium supplementation is balanced in both arms, significantly increases the risk of hypercalciuria by 64%. Since vitamin D may increase kidney stone formation through an increase in urine calcium excretion, the evaluation of urinary calcium excretion after vitamin D supplementation is a major concern. Only two studies investigated the effect of nutritional vitamin D on urinary calcium excretion on a prospective basis [70,71]. Leaf et al. prescribed ergocalciferol to 29 stone formers with relatively low 25-hydroxyvitamin D serum levels at baseline and concluded that a limited course of vitamin D repletion does not aggravate the mean urinary calcium excretion, although a subset of individuals may have an increase [70]. Johri et al. recently described an overall rise in 24-h urine calcium excretion following cholecalciferol supplementation in kidney stone formers, which failed to reach statistical significance (*p* = 0.06) [71]. Six out of 26 initially normocalciuric stone formers developed hypercalciuria; and six out of the nine patients who became vitamin D replete (serum level > 30 ng/mL) were hypercalciuric after supplementation.

Taken together, these results suggest that some predisposed individuals may potentially develop or worsen hypercalciuria after vitamin D repletion. The widespread prescription of vitamin D supplements in the general population might therefore increase the risk of kidney stone formation in patients prone to developing hypercalciuria.

## 8. Randall’s Plaque and Calcium Stone Formation: A Link with Vitamin D Prescription?

Alexander Randall described, in 1936, that calcium phosphate deposits at the tip of renal papillae, the Randall’s plaques, were at the origin of kidney stones in around 20% of kidneys [7]. Several authors found a similar occurrence of calcium phosphate deposits in Caucasians from various countries [72,73]. These plaques form in the interstitial tissue, spread and break the urothelium at later stages, promoting heterogeneous nucleation of calcium oxalate crystals and calcium oxalate stone formation. Randall’s plaques, which are made of calcium phosphate are therefore the first step toward kidney stone formation; they are made of monohydrate and dihydrate calcium oxalate in most cases and are a matter of concern since the proportion of calcium oxalate stones formed on plaques has increased during the past decades, at least in some countries [6]. Over the past half century, the occurrence of calcium stones developed from Randall’s plaques dramatically increased: up to 75–80% of calcium stones in the US, and 55–60% of calcium stones in France, where 48.8% of spontaneously passed calcium oxalate calculi exhibit fragments of a papillary Randall’s plaque [10,74]. It has been reported that Randall’s plaque formation was related to hypercalciuria and that coverage of papilla by Randall’s plaque is related to calcium excretion (hypercalciuria) and low volume of diuresis [9,75]. Moreover, stone formation was linked to papillary surface coverage by Randall’s plaques [76]. Since hypercalciuria is a determinant of Randall’s plaque formation, probably by increasing calcium phosphate supersaturation in kidney interstitial tissue, vitamin D could, in theory, influence plaque growth and formation [9]. Nevertheless, monohydrate calcium oxalate stones developed on plaques are mainly related to low diuresis and an increased urine oxalate concentration, evidencing that the processes leading to plaque and stone formation, respectively, may differ [77]. Of notice, plaque may form in kidney tissue many years before kidney stones, sometimes during childhood, and urine composition may have changed with time.

We still ignore whether vitamin D prescription or vitamin D metabolite serum levels could influence Randall’s plaque formation. We compared serum and urine biochemistry from calcium oxalate stone formers, with and without Randall’s plaque at the origin of the stones. We did not find evidence for any difference in vitamin D metabolites between the two groups, but patients affected by plaques had higher serum calcium levels, higher osteocalcin serum levels, lower phosphate excretion levels and a trend toward decreased parathyroid hormone levels—all features compatible with an increased sensitivity to vitamin D [6]. Nevertheless, VDR polymorphisms could not explain the phenotype observed. Whether individual sensitivity to vitamin D could promote Randall’s plaque formation needs to be confirmed by additional studies. Patients who form stones from Randall’s plaques are younger, raising concerns about a potential role of vitamin D prescribed during infancy in Randall’s plaque development, whereby stones are formed years or decades later [6].

## 9. Summary

There is growing evidence that cholecalciferol administration or 25-hydroxyvitamin D serum levels, in the higher ranges, may increase urinary calcium excretion and kidney stone formation in predisposed individuals or specific groups of patients. Over the past few decades, the observation of an association between “low” levels of circulating 25-hydroxyvitamin D serum levels and a broad spectrum of diseases has been at the origin of a dramatical increase in the prescription of vitamin D. However, most of studies evaluating the effects of vitamin D administration, including studies dedicated to bone fractures, did not demonstrate a significant benefit of vitamin D [77,78,79,80].

In parallel, the risk of developing kidney stones, especially when vitamin D intakes are combined with calcium prescription, should be taken into consideration. It seems likely that some predisposed individuals, possibly prone to transforming 25-hydroxyvitamin D into calcitriol, with a reduced capacity for degrading calcitriol, or those who are more “sensitive” to vitamin D signalling, are more at risk of developing kidney stones in response to vitamin D prescription; however, the predisposing alleles have not been identified yet. The specific question regarding the Randall’s plaque should be taken into consideration. A large and possibly increasing number of stones are due to these plaques, whose formation depends on calcium phosphate supersaturation at the tip of the renal papilla. Randall’s plaques precede the development of kidney stones and are more frequently observed in children nowadays, raising concerns about a potential role of vitamin D prescribed during infancy in Randall’s plaque development, in turn leading to the formation of stones years or decades later. The identification of patients at risk of developing kidney stones in response to vitamin D prescription will be a medical challenge in the future.
